# Wnt/β-Catenin Signaling Activation Induces Differentiation in Human Limbal Epithelial Stem Cells Cultured Ex Vivo

**DOI:** 10.3390/biomedicines11071829

**Published:** 2023-06-26

**Authors:** Jovana Bisevac, Kirankumar Katta, Goran Petrovski, Morten Carstens Moe, Agate Noer

**Affiliations:** 1 Center for Eye Research and Innovative Diagnostics, Department of Ophthalmology, Oslo University Hospital, P.O. Box 4956 Nydalen, 0424 Oslo, Norway; goran.petrovski@medisin.uio.no (G.P.); m.c.moe@medisin.uio.no (M.C.M.); agate.noer@medisin.uio.no (A.N.); 2 Institute of Clinical Medicine, Faculty of Medicine, University of Oslo, 0318 Oslo, Norway; 3 Department of Immunology, Oslo University Hospital, Hf Rikshospitalet, 0424 Oslo, Norway

**Keywords:** limbal epithelial stem cells, Wnt/β-catenin signaling, stemness, proliferation, differentiation

## Abstract

Human limbal epithelial stem cells (hLESCs) continuously replenish lost or damaged human corneal epithelial cells. The percentage of stem/progenitor cells in autologous ex vivo expanded tissue is essential for the long-term success of transplantation in patients with limbal epithelial stem cell deficiency. However, the molecular processes governing the stemness and differentiation state of hLESCs remain uncertain. Therefore, we sought to explore the impact of canonical Wnt/β-catenin signaling activation on hLESCs by treating ex vivo expanded hLESC cultures with GSK-3 inhibitor LY2090314. Real-time qRT-PCR and microarray data reveal the downregulation of stemness (*TP63*), progenitor (*SOX9*), quiescence (*CEBPD*), and proliferation (*MKI67*, *PCNA*) genes and the upregulation of genes for differentiation (*CX43*, *KRT3*) in treated- compared to non-treated samples. The pathway activation was shown by *AXIN2* upregulation and enhanced levels of accumulated β-catenin. Immunocytochemistry and Western blot confirmed the findings for most of the above-mentioned markers. The Wnt/β-catenin signaling profile demonstrated an upregulation of *WNT1*, *WNT3*, *WNT5A*, *WNT6*, and *WNT11* gene expression and a downregulation for *WNT7A* and *DKK1* in the treated samples. No significant differences were found for *WNT2*, *WNT16B*, *WIF1*, and *DKK2* gene expression. Overall, our results demonstrate that activation of Wnt/β-catenin signaling in ex vivo expanded hLESCs governs the cells towards differentiation and reduces proliferation and stem cell maintenance capability.

## 1. Introduction

The corneal epithelium is a dynamic, transparent, multilayered structure composed of non-keratinized human corneal epithelial cells (hCECs) that acts as a physical, chemical, and immune barrier to the cornea and the rest of the eye [1]. The superficial layer of the human corneal epithelium is rapidly lost by apoptosis and regularly shed off into the tear film. The consecutive replacement of diminished hCECs is governed by adult stem cells located in the basal epithelial layer of the limbus at the junction between the corneal and conjunctival/scleral tissue of the eye [2,3,4]. Human limbal epithelial stem cells (hLESCs) are found in their unique stem cell niches in the limbal crypts [5] of the palisades of Vogt [6,7]. There, specific niche cells and extracellular matrix elements [8] allow the hLESCs to remain reversibly quiescent [4]. It is reported that the combined expression of several genes features the signature of hLESCs [9], such as the presence of the putative stem cell marker ΔNp63α, the marker of quiescence CEBP/δ, as well as the absence of differentiation markers such as Cx43, KRT3, KRT12, and others [10,11,12,13,14]. hLESCs can divide symmetrically, giving rise to two equal daughter stem cells [15]. In addition, hLESCs perform asymmetrical cell division to produce one stem cell and a more differentiated progeny, a transient amplifying cell (TAC). TACs further divide and gain more distinct properties with each division until they lose their proliferation potential and differentiate into mature hCECs [14,16,17,18]. This event is followed by simultaneous migration of the TACs from the basal limbus to the superficial central corneal layers [18,19]. hLESC proliferation rate increases rapidly during injuries to repair the damaged cornea [4]. Proliferation and stem cell pool expansion are the first steps in such states. The second is consecutive differentiation to progeny/TAC production and maturation, followed by a decrease in the stem cell pool. Thus, the counterbalance between hCEC production and corneal reconstruction is sustained by hLESC preservation in the limbal crypts [9,18].

Reduced number and/or function of hLESCs, so-called limbal stem cell deficiency (LSCD), leads to the inability to maintain an intact corneal epithelium [20]. The cultivated limbal epithelial stem cell transplantation (CLET) of ex vivo expanded cells from a small limbal biopsy of the contralateral eye on the 3T3 fibroblast feeder layer [21] or on the human amniotic membrane (HAM) [10] is one of the most common treatments for LSCD. Still, treatment of LSCD has a lot of challenges, such as etiology and pathology heterogeneity, no standard surgical and culture protocols, and a lack of objective measurement of treatment outcome. Recently, novel biotechnology strategies for LSCD are being tested, such as gene therapy and gene editing, microRNAs, nanotransporters, novel small molecules, and induced pluripotent stem cell (iPSC) therapy [11]. Hence, the progress of treatment procedures requires the identification of key stem-cell-related molecules and understanding the signaling pathways governing hLESC fate from stemness, quiescence, and the self-renewal state to hLESC/TAC proliferation, migration, differentiation, and stratification.

Wnt/β-catenin signaling, an evolutionary conserved molecular pathway involved in stem cell fate determination [22,23], is highly expressed in limbal tissue compared to the cornea and conjunctiva [24]. The canonical Wnt/β-catenin dependent and the non-canonical/β-catenin signaling-independent pathways are two principal Wnt signaling extensions [25]. Canonical Wnt/β-catenin is fundamental to stem cell fate, inducing quiescence or self-renewal; furthermore, it is critical for TAC proliferation and differentiation. Tissue renewal is attenuated when Wnt/β-catenin signaling is inhibited [26]. Wnt/β-catenin signaling also depends on the engagement of both Frizzled receptors and Lrp5/6 transmembrane co-receptors forming a complex that inhibits glycogen synthase kinase 3 (GSK-3) and the establishment of a destruction complex that targets β-catenin through phosphorylation for proteasomal degradation [27]. Non-phosphorylated β-catenin can associate with Tcf/Lef nuclear transcription factors and initiate the transcription of Wnt/β-catenin target genes. Canonical Wnt signaling is maintained by 19 Wnt proteins, 10 Frizzled (Fzd) receptors, 4 Dickkopf (Dkk) inhibitors, and other Wnt inhibitors [26,27]. 

As mentioned above, the ex vivo expansion and cultivation of hLESCs from limbal biopsies and, thereafter, transplantation (CLET) is one of the most commonly used methods for the treatment of LSCD. However, most of the previous studies have focused their research on Wnt/β-catenin activation on hLESCs isolated as single-cell cultures, and the previous data regarding the Wnt/β-catenin signaling in ex vivo expanded hLESCs from limbal biopsies are scarce [28,29,30,31]. Ex vivo expansion of hLESCs from limbal biopsies prior to transplantation is an alternative method and also the preferred method of isolation and cultivation of hLESCs at the Department of Ophthalmology, Oslo University Hospital. Therefore, we wanted to explore whether the activation of Wnt/β-catenin signaling increases the stemness and proliferation of the hLESCs isolated by this culture method.

In the present study, we aimed to test the effect of the small molecule LY2090314, a GSK-3 inhibitor and Wnt/β-catenin activator, on the cell fate of hLESCs expanded from healthy limbal biopsies cultivated ex vivo. In addition, we searched for particular Wnt signaling ligands and pathway molecules upon Wnt/β-signaling activation in hLESCs.

## 2. Materials and Methods

All tissue collection complied with the Guidelines of the Helsinki Declaration. The laboratory procedures and tissue harvesting were approved by the Regional Committee for Medical and Health Research Ethics, Norway (No 2017/418). Unless stated otherwise, all reagents were purchased from Merck (Darmstadt, Germany).

### 2.1. Limbal Biopsies and Human Limbal Epithelial Stem Cell (hLESC) Cultures

The preparation of limbal biopsies and in vitro expansion of hLESCs was performed according to the standard protocol [30]. Corneoscleral rings from human donors, remaining after corneal transplantations, were used for hLESC isolation and harvesting in vitro. Each ring was divided into 12 equal pieces of limbal biopsies encompassing nearly 1 mm of peripheral cornea and 1 mm of the adjacent sclera to ensure the whole limbal area was included. They were further rinsed in DMEM/F12 medium (31331028, Invitrogen, Carlsbad, CA, USA) containing 100 U/mL penicillin and 100 μg/mL streptomycin (P4333). After that, the biopsies were treated with 2.4 U/mL Dispase II (4942078001, Roche Diagnostics, Mannheim, Germany) in Mg^2+^ and Ca^2+^ free Hank’s Balanced Salt Solution (HBSS, H8848) for 10 min at 37 °C, followed by washing in Fetal Bovine Serum (FBS, F2442). The limbal biopsies were plated with the epithelial side down on six-well TC-Treated Multiple Well Plates (CLS3516, Corning Inc., New York, NY, USA). The biopsies were submerged in complex medium (COM) containing DMEM/F12 (31331028, Invitrogen, Carlsbad, CA, USA), 100 U/mL penicillin, and 100 μg/mL streptomycin (P4333), 2.5 μg/mL Amphotericin B (A2942), 5% FBS (F2442), 2 ng/mL epidermal growth factor (E9644), 5 μg/mL insulin, 5 ng/mL sodium selenite and 5 μg/mL transferrin (l1884), 30 ng/mL cholera toxin A subunit from *Vibrio cholerae* (C8180), 0.5% dimethyl sulfoxide (DMSO, D2650), and 15 μM hydrocortisone (H0888). Once the biopsies attached to the well, they were completely immersed in COM and incubated at 37 °C, 5% CO_2_, and 95% air for the following nine days. The culture medium was changed every 2–3 days.

### 2.2. Small Molecule LY2090314 Treatment of HLESCs

The hLESCs proliferated and expanded from the limbal biopsies in COM on the six-well plates for nine days. Thereafter, the COM was removed and replaced by COM containing 1 nM, 2 nM, 5 nM, and 10 nM of LY2090314, when applicable. One to three wells per condition per donor (*n* = 3–6) were used for further analysis. In addition, DMSO reciprocal to the DMSO concentration in cultures treated with 10 nM LY2090314 was added to individual wells as controls. The DMSO dilution of 10 nM LY2090314 concentration in COM medium was 1:10,000. Images from primary hLECS cultures were acquired using a light microscope (Nikon TS100, Nikon, Tokyo, Japan).

### 2.3. Cytotoxicity Assay

Once the primary hLESCs growing out of the limbal biopsies reached confluence, they were treated with 0.25% Trypsin-EDTA (T3924) and seeded on 24-well plates (Corning Inc., New York, NY, USA) at 6 × 10^5^ cells/well density and incubated for 24 h in a humidified 5% CO_2_ incubator at 37 °C. After the cell cultures were washed with COM, the medium was replaced with fresh COM or COM containing 1 nM, 2 nM, 5 nM, and 10 nM LY2090314 and DMSO-containing control. The medium was collected and changed on days 1, 3, 6, and 9. A CytoTox96 Non-Radioactive Cytotoxicity assay (G1780, Promega Corporation, Madison, WI, USA) was used; LDH release was measured from the collected medium according to the manufacturer’s instructions. The colorimetric values of three replicates were monitored at a wavelength of 490 nm on a Victor^3^ multilabel plate reader. 

### 2.4. RNA Isolation and Real-Time Quantitative RT-PCR (RT-qRT-PCR)

RNA isolation for Real-Time qRT-PCR analysis was performed from the paired individual wells of the six-well plate from five human donors (*n* = 5) containing: (1) untreated, control hLESCs cultured in COM and (2) treated hLESCs cultured in COM containing 5 nM LY2090314 harvested for nine days. The medium was removed and replaced by a fresh medium every three days. 

Total RNA was isolated from the non-treated and treated hLESCs using 1–3 individual wells of six-well plates for each condition. RNA isolation was carried out using an RNeasy Micro Kit (Qiagen, Hilden, Germany) and quantified via spectrophotometry (Nanodrop, Wilmington, Germany). Reverse transcription (RT) was performed using a high-capacity cDNA Reverse Transcription Kit (Applied Biosystems, Abingdon, UK) with 1 μg total RNA per 20 μL RT reaction, followed by cDNA dilution to 2 ng/μL using 180 μL RNase/DNase free water (Thermo Fisher Scientific, Waltham, MA, USA). The StepOneplus Real-Time qRT-PCR (Applied Biosystems) and TaqMan Gene expression assays, including predesigned primers/probes (Applied Biosystems, Supplementary Table S1), were used for comparative relative quantification. The thermocycling conditions for Real-Time qRT-PCR were 95 °C for 10 min, followed by 40 cycles of 95 °C and 60 °C for 1 min. All samples were run in triplicates (2.5 ng cDNA in a total volume of 12.5 μL). The 2^−ΔΔCt^ comparative method was used to interpret the fold change (FC) in gene expression and standardize results to the endogenous reference gene, 18S values. 

### 2.5. Microarray and Data Analysis

The microarray analyses were performed at the Affymetrix Core Facility, Ullevål, Oslo University Hospital, South-Eastern Norway Regional Health Authority. Affymetrix GeneChip Clariom S Human (Affymetrix, Santa Clara, CA, USA) was used for the analyses. 

We subjected 150 ng of total RNA to the GeneChip WT PLUS Reagent Kit, following the manufacturer’s protocol for whole-genome gene expression analysis (Affymetrix, Santa Clara, CA, USA). The labeled and fragmented single-stranded cDNAs were hybridized to GeneChip Clariom S Human (Affymetrix, Santa Clara, CA, USA). The arrays were washed and stained using an FS-450 fluidics station (Affymetrix, Santa Clara, CA, USA). The signal intensities were detected using a Hewlett Packard Gene Array Scanner 3000 7G (Hewlett Packard, Palo Alto, CA, USA). The scanned images were processed using AGCC (AffymetrixGeneChip Command Console) software. For statistical analysis of the gene expression, the Affymetrix CEL files (containing probe intensities) were imported into Partek Genomics Suite software (Partek Inc., Chesterfield, MO, USA). A robust microarray analysis (RMA) yielding normalized log_2_ transformed signal intensities was applied for normalization (http://bip.weizmann.ac.il/toolbox/overview/Partek_Users_Guide.pdf) accessed 12 September 2020. Gene transcripts with maximal signal values of less than 5 (log_2_ values) across all arrays were removed to filter out low and non-expressed genes. The differentially expressed transcripts between groups “treated vs. control” were identified using two-way ANOVA as implemented in Partek Genomics Suite. The results are expressed as FC. Genes with FC ≥ 1.5 and a *p*-value < 0.05 were regarded as significantly regulated.

The Affymetrix gene expression data were deposited in NCBI’s Gene Expression Omnibus [32] and are accessible through GEO Series accession number GSE234403 at (https://www.ncbi.nlm.nih.gov/geo/query/acc.cgi?acc=GSE234403, accessed on 29 April 2023).

### 2.6. Immunocytochemistry (ICC)

The primary cultures growing out from limbal biopsies were washed with Dulbecco’s Phosphate-Buffered Saline (DPBS, 14190-144, Thermo Fisher Scientific, Waltham, MA, USA) and fixed with 4% formaldehyde for 15 min at room temperature, then washed again 2 times with DPBS and permeabilized with 0.5% Triton X-100 (93443) for 15 min. After that, the cells were blocked with 5% BSA in 0.1% Triton X-100/DPBS for 30 min. The primary antibody was dissolved in 0.5% BSA/0.1% Triton X-100/DPBS and incubated for 3 h at room temperature. The following primary antibodies were used: proliferation marker Ki-67 (rabbit monoclonal, 1:200 dilution, RM-9106-S, Thermo Scientific, Waltham, MA, USA), progenitor markers: tumor protein p63 alpha (p63α, rabbit polyclonal, 1:200, 4892S, Cell Signaling, Danvers, MA, USA), SRY-Box Transcription Factor 9 (SOX9, rabbit monoclonal, 1:200 dilution, 82630, Cell Signaling, Danvers, MA, USA), and a differentiation marker: connexin-43 (Cx43, rabbit polyclonal, 1:300, C6219). After incubation, the slides were washed 3 times with DPBS for 10 min. Incubation was continued with the appropriate animal secondary antibody type: Cy3^®^ goat anti-rabbit IgG (rabbit monoclonal, 1:500 dilution, A10520, Abcam, Cambridge, UK) for samples stained with Ki-67, p63α and SOX9, and Alexa Fluor^®^ 488 donkey anti-rabbit IgG (1:500 dilution, rabbit monoclonal, A21206, Abcam, Cambridge, UK) for antibody stained with Cx43. Next, the nuclear staining was performed with Hoechst 33342 staining (1:2000 dilution, H3570, Thermo Fisher Scientific, Waltham, MA, USA) for 15 min at room temperature. Finally, the samples were washed 3 times with DPBS for 10 min and mounted (ProLong™ Diamond Antifade Mountant P36970, Thermo Fisher Scientific, Waltham, MA, USA) onto slides. The fluorescence was recorded using a ZEISS Axio Imager M1 fluorescence microscope (ZEISS, Obercochen, Germany). Nuclear antibody positivity (p63α, SOX9, and Ki67) was counted by three independent individuals. 

### 2.7. Western Blot (WB)

Equal protein concentrations (30 μg) previously measured using albumin standards (23235, Thermo Scientific, Waltham, MA, USA) were separated by size using electrophoresis on 4–15% Criterion TGX Gels (Bio-rad laboratories, Inc. Hercules, CA, USA). The proteins were then further transferred from the gels to PVDF membranes by semidry electrotransfer. The membranes were blocked with 5% dry milk or 3% BSA in Tris-Buffered Saline/TBS)/0.1% Tween buffer (28360, Thermo Fisher Scientific, Waltham, MA, USA) for 1 h at room temperature. The 1× TBS/0.1% Tween solution was also used as a washing buffer and a diluent for the antibodies. Thereafter, the membranes were incubated with anti-Cx43 polyclonal rabbit (1:1000, C6219) or anti-β-catenin (1:1000, 9562, Cell Signaling Technology, Danvers, MA, USA) overnight at 4 °C. The membranes were washed three times for 5 min, followed by 1 h of incubation at room temperature with the same-origin horseradish peroxidase-linked whole secondary antibody (1:5000, GENA934/GENA931, Global Life Sciences Solutions Operations, Buckinghamshire, UK). After washing, the protein–antibody complexes were detected using the enhanced chemiluminescent assay after adding the substrate (34577, Thermo Fisher Scientific, Waltham, MA, USA). The band intensities given by Cx43 were normalized to Vinculin using a monoclonal anti-Vinculin antibody produced in mouse (1:100,000, V9131), and the band intensities given by β-catenin were normalized to β-actin using anti-β-actin antibody produced in mouse (1:5000, A5441, Merck Life Science, Darmstadt, Germany) and the appropriate secondary antibody. 

### 2.8. Edu Cell Proliferation Assay

The primary cell cultures were washed with DPBS and trypsinized in 0.25% Trypsin-EDTA for 10 min, blocked with FBS dissolved in DMEM/F-12, and centrifuged for 10 min on 302× *g* at 22 °C. After cell counting, the cells were plated on coverslips in 12-well TC-Treated Multiple-Well Plates (CLS3516, Corning Inc., New York, NY, USA). The coverslips were previously coated with poly-L-lysine (P4707) for 1 h. Thereafter, in the different paired individual wells of the control and treated cells from three individual donors, 10 μM EdU (Click-iT™ EdU Cell Proliferation Kit for Imaging, Alexa Fluor™ 488 dye, C10337, Invitrogen™, Thermo Fisher Scientific, Waltham, MA, USA) was added for 1 day, from day 1 to day 3, from day 4 to day 6, and from day 6 to day 9, so that the EdU could incorporate into the DNA during cell replication during these time intervals. Thereafter, the cultured cells were washed 3 times with PBS containing 3% BSA and fixed with 4% formaldehyde for 20 min, then washed again with 3% BSA in PBS and permeabilized with 0.5% Triton X-100 in PBS for 20 min. Ultimately, the cells were stained with a reaction cocktail that was made according to the manufacturer’s instructions for 30 min protected from light. Then, the nuclei were stained with 5 µg/mL Hoechst 33342 solution. The coverslips with cells were mounted on slides and fluorescent images were taken accordingly. Quantification of the EdU-positive cells was carried out the same as for previously mentioned markers. 

### 2.9. Statistical Analysis

Descriptive statistical methods, mean ± SEM, were used to describe the gene and protein expression values from the available replicates of the same donors and group of donors. The statistical analyses were performed using Prism 8.3.0 (GraphPad, San Diego, CA, USA). After the data were tested for normal distribution (Shapiro–Wilk test), the significant differences between the two groups were determined using a parametric unpaired two-sample t-test or nonparametric Mann–Whitney *U* test, and *p* values < 0.05 were considered to be statistically significant.

## 3. Results

### 3.1. Wnt/β-Catenin Signaling Activation Affects Cell Morphology in hLESC Cultures

**Figure 1:**
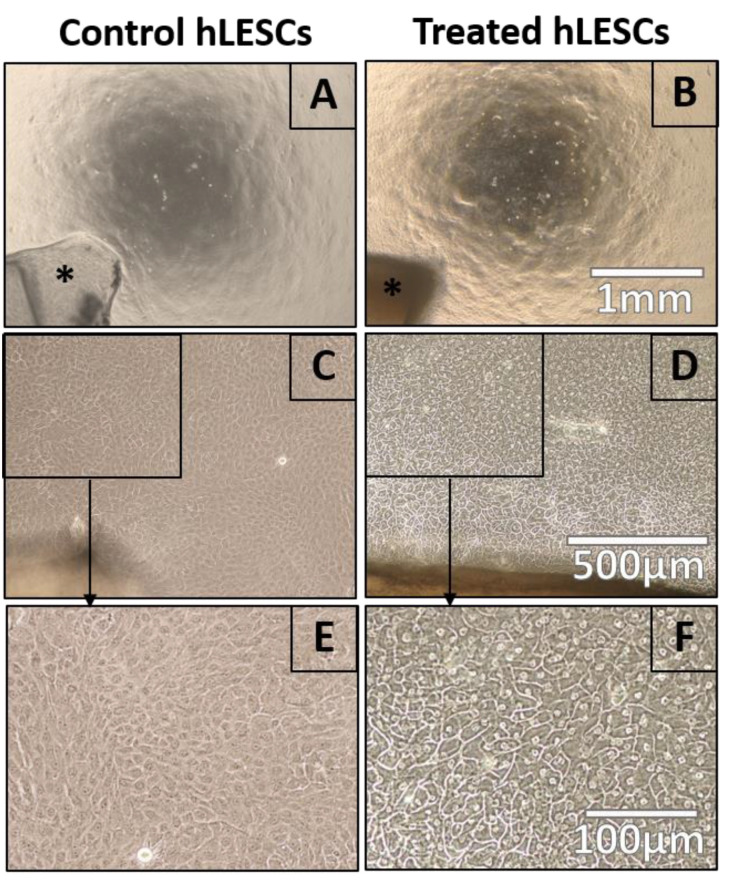
Light microscopy demonstrating morphology of the primary hLESC cultures upon treatment with 5 nM LY2090314, a GSK-3 inhibitor, and Wnt/β-Catenin signaling activator. Primary hLESCs were growing out of limbal biopsies (black star) for nine days in COM medium. Thereafter, the concentration of 5 nM LY2090314 was added to individual wells for additional nine days. Control (non-treated) hLESC cultures (**A**,**C**,**E**) appeared morphologically different from 5 nM LY2090314 treated hLESC cultures (**B**,**D**,**F**) regarding enlarged cell size, membrane thickening, and increased nucleus-to-cytoplasm (N:C) ratio. Scale bars are the same for all images in the same row.

The primary hLESC cultures treated with LY2090314 appeared to have a changed morphology, cell size enlargement, and a more pronounced nucleus and nucleoli when compared to controls (Figure 1). The cell membrane also appeared thicker in treated hLESC cultures compared to controls. The morphological changes were most notable in the cells treated with 5 nM LY2090314 (Figure 1 and Figure S1D,J) after nine days of treatment, whereas the 10 nM LY2090314 concentration seemed to be harmful to the cell cultures, with the presence of many detached and dead cells in addition to debris floating in the medium (Supplementary Figure S1E,K). Cells cultured in the DMSO-containing control medium were morphologically similar to the controls (Supplementary Figure S1F,L).

### 3.2. hLESC Toxicity upon Treatment with Increasing Concentrations of Selective Small-Molecule LY2090314

**Figure 2:**
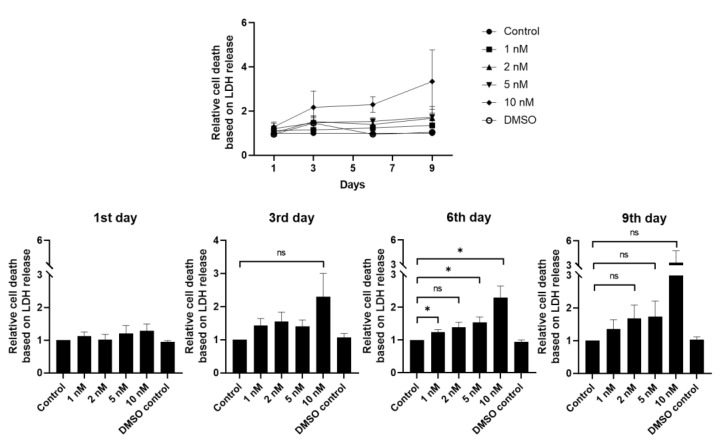
Relative cell death based on the LDH release upon treatment with increasing dose-dependent concentrations of selective small-molecule LY2090314. LDH release was measured from supernatants in the hLESC cultures treated with 1 nM, 2 nM, 5 nM, and 10 nM LY2090314 concentrations for nine days and compared to control hLESC cultures. The LDH was measured on days 1, 3, 6, and 9. All data from three donors (*n* = 3) are presented as mean ± SEM. *: *p <* 0.05, ns: not significant.

To examine the possible cytotoxicity of LY2090314, we measured LDH release from hLESCs treated with increasing drug concentrations at days 3, 6, and 9 of treatment. LDH release in treated cells was not statistically significant after three days of treatment compared to control/non-treated, confirming no passive leakage from the cells during this period. On day 6, LDH measurement demonstrated higher release in hLESCs upon 1 nM, 5 nM, and 10 nM of treatment than in control (*p* < 0.05), whereas this measurement was not higher in hLESCs upon treatment with 2 nM concentration and DMSO control. However, no statistical difference was found after nine days of treatment in the LDH release between treated and control hLESCs (Figure 2). 

### 3.3. Real-Time qRT-PCR Identified Wnt/β-Catenin Signaling Activation in hLESC Cultures and Upregulation of Genes for Differentiation and Downregulation of Genes for Limbal Epithelial Stemness Maintenance and Proliferation

**Figure 3:**
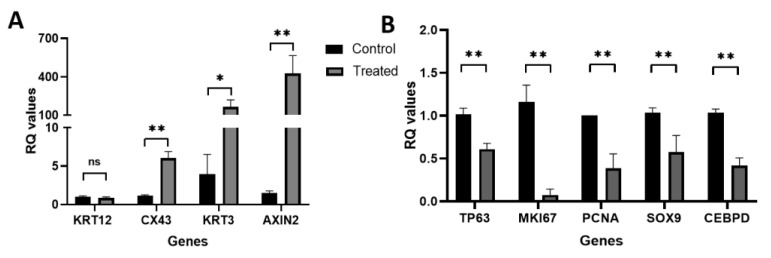
Gene expression of primary hLESCs upon Wnt/β-catenin activation. hLESCs were treated with 5 nM LY2090314 for nine days and compared to untreated control cells. Real-time qRT-PCR revealed the difference in expression of genes for (**A**) differentiation (*CX43*, *KRT3*, and *KRT12*), pathway control gene *AXIN2*, and genes for (**B**) stemness and quiescence (*TP63* and *CEBPD*), progenitor (*SOX9*), and proliferation (*MKI67*, *PCNA*) of treated hLESCs in comparison to control hLESCs. The data from five donors (*n* = 5) are presented as mean ± SEM, *: *p <* 0.05, **: *p <* 0.01, ns: not significant.

Gene analysis of hLESC cultures treated for nine days with 5 nM LY2090314, compared to the controls, revealed statistically significant upregulation of genes for epithelial differentiation, such as *CX43* (mean RQ and FC: 6-fold, *p* < 0.01), *KRT3* (165.6-fold, *p* < 0.05), and pathway control gene *AXIN2* (462-fold, *p* < 0.01), whereas significance was not found in cytokeratin 12 (*KRT12*, 0.84-fold, *p* = 0.4) gene expression (Figure 3A). In addition, the genes regulating stem cell maintenance and self-renewal, *TP63* and CCAT/enhancer binding protein delta (*CEBPD*), and those regulating limbal basal/progenitor cell maintenance, *SOX9* as well as proliferation, *MKI67*, and proliferation cell nuclear antigen (*PCNA*) were significantly downregulated in treated hLESC cultures (mean RQ and FC of treated hLESC for *TP63*: 0.6-fold, *MKI67*: 0.08-fold, *PCNA*: 0.4-fold, *SOX9*: 0.6-fold and *CEBPD*: 0.4-fold, *p* < 0.01) (Figure 3B). 

### 3.4. Wnt Signaling mRNA Expression Profile in Primary hLESC Cultures upon Treatment with LY2090314

**Figure 4:**
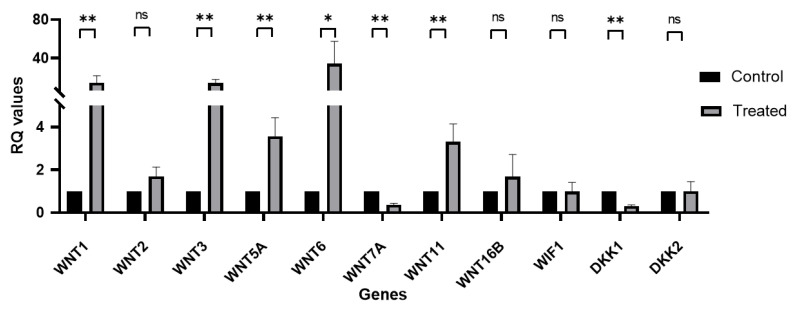
Wnt signaling gene expression profile in primary hLESCs upon treatment with LY2090314. Wnt signaling gene expression in primary cultured hLESCs treated with 5 nM LY2090314 for nine days was compared to control hLESC cultures. Significant upregulation was found for *WNT1*, *WNT3*, *WNT5*, *WNT6*, and *WNT11*, while significant downregulation was revealed for *WNT7A* and *DKK1*. No differences in expression were found for *WNT2*, *WNT16B*, *WIF1*, and *DKK2* when comparing the treated and untreated control hLESCs. The data from five donors (*n* = 5) are presented as mean ± SEM, *: *p <* 0.05, **: *p <* 0.01, ns: not significant.

To comprehensively characterize Wnt signaling expression upon GSK-3β inhibition in hLESCs, we further analyzed the gene expression of hLESCs treated with 5 nM LY2090314 for nine days and compared it to control hLESCs. Significant upregulation was found for the *WNT1*, *WNT3*, *WNT5A*, *WNT6*, and *WNT11* genes (mean RQ and FC: 14.5-fold, 14.5-fold, 3.6-fold, 34.2-fold, 3.3-fold, respectively), whereas significant downregulation was found for *WNT7A* and *DKK1* (mean RQ and FC: 0.7-fold and 0.3-fold change, respectively). No statistically significant difference in the mRNA levels and hence gene expression could be found for *WNT2*, *WNT16B*, *WIF1*, and *DKK2* (mean RQ and FC: 1.7-fold, 1.7-fold, 1.0-fold, and 1.0-fold, respectively) (Figure 4). 

### 3.5. Microarray Data Analysis of Primary hLESC Cultures upon Treatment with 5 nM Concentration of LY2090314 Compared to Untreated Controls

**Figure 5:**
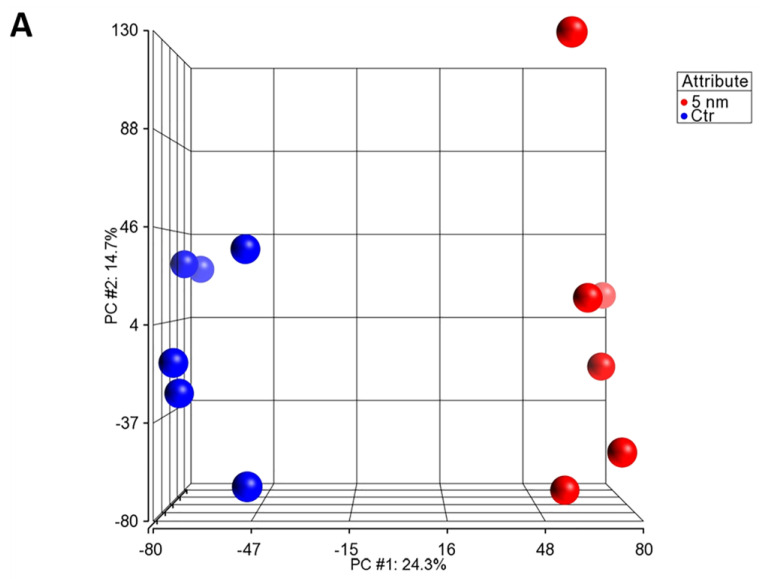

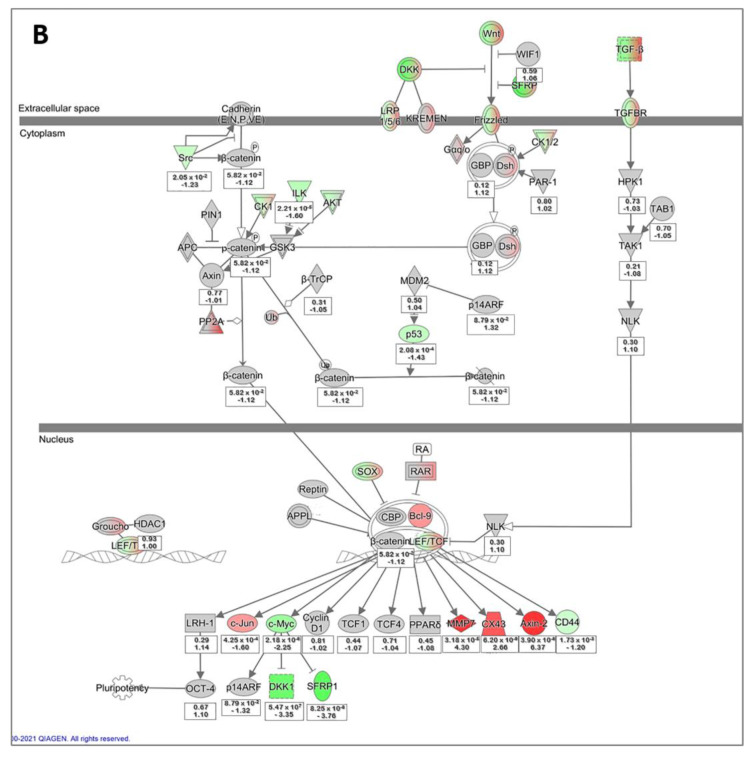
Microarray data analysis of the hLESCs treated with Wnt/β-catenin activator and control hLESCs. (**A**) The principal component analysis (PCA) plot demonstrates two different clusters: the control (untreated) samples as red ellipses vs. samples treated with 5 nM LY2090314 as blue ellipses. Samples are matched pairs of the same donors. (**B**) Ingenuity pathway analysis (IPA) shows changes in the expression of genes involved in Wnt/β-catenin signaling in hLESCs treated with LY2090314 compared to control hLESCs. Upregulated genes are marked with red and pink figures, while downregulated genes are marked with green figures. The stronger color intensity demonstrates a greater change in expression. Data from six donors (*n* = 6) presented with *p*-values given below the figures, whereas the fold changes (FCs) in gene expression were found to be below the *p*-values.

The six donors with matched individual pairs of 1–3 individual wells of control or treated hLECS cultures were clustered in an unsupervised fashion according to the gene expression profiles as shown in the PCA plot (Figure 5A). A total of 14 genes involved in the Wnt/β-catenin canonical pathway demonstrated significant differential expression when adjusting the FC barrier to ±2 at a nominally significant *p*-value (*p* < 0.05) and multiple testing correction (FDR < 0.05). Nine genes were expressed at higher FC levels (*ACVR1C*, *AXIN2*, *FZD7*, *GJA1*, *MMP7*, *PPP2R28*, *TCF7*, *TGFB3*, *and WNT5A*) and five genes were expressed at lower FC levels (*DKK1*, *MYC*, *SFRP1*, *TGFB2*, *WNT7A*) in the treated hLESC cultures. Table 1 summarizes the list of differentially expressed genes as well as adjusted *p*-values and FCs in treated- compared to non-treated samples. Wnt/β-catenin canonical pathway genes that are differentially expressed upon treatment with GSK3 inhibitor LY2090314 are rendered per IPA legend (Figure 5B).

### 3.6. Immunocytochemistry and Western Blot Analysis of 5 nM LY2090314-Treated hLESC Cultures Revealed Higher Levels of β-Catenin and Cx43 Protein Expression with Low Levels of Ki-67, SOX9, and p63α

**Figure 6:**
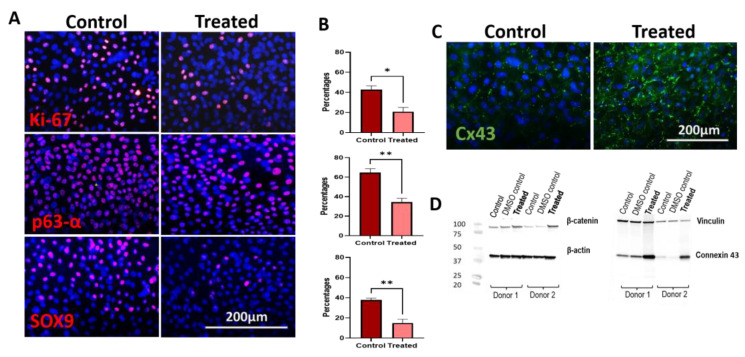
Proliferation, progenitor, and differentiation marker expression in primary hLESCs upon Wnt-β activation. (**A**) Immunocytochemistry of proliferation marker Ki-67 and progenitor markers, p63α and SOX9, in control, untreated hLESC cultures and nine-days-treated hLESC cultures with 5 nM LY2090314 demonstrating (**B**) statistically significant downregulation of respective markers in the treatment group. Data from three donors (*n* = 3) are presented as mean ± SEM, *: *p <* 0.05, **: *p <* 0.01. (**C**) Immunocytochemistry of differentiation marker, Cx43 in control hLESC cultures vs. nine-days-treated hLESCs revealed higher expression of Cx43 in the treated group (*n* = 3) (**D**). Western blot demonstrated higher expression levels of β-catenin and Cx43 protein expression in the treatment group (*n* = 2).

The proliferation marker Ki-67 was statistically downregulated in treated samples compared to controls quantified as percentages (control vs. treated, 42.82 ± 3.73% vs. 20.76 ± 4,34%, *p* = 0.02 < 0.05), as well as progenitor markers: p63α (64.56 ± 4.06 vs. 34.34 ± 3.93, *p* < 0.01) and SOX9 (37.77 ± 1.89 vs. 15.01 ± 3.73, *p* < 0.01) (Figure 6A,B). On the other side, ICC and WB revealed higher protein expression of the differentiation protein Cx43 in the treated group (Figure 6C,D). The higher expression levels of β-catenin in treated cells were validated by WB (Figure 6D).

### 3.7. Edu Proliferation Assay Demonstrates Lower Proliferation upon Wnt/β-Catenin Signaling Activation in Ex Vivo Expanded hLESC Cultures

**Figure 7:**
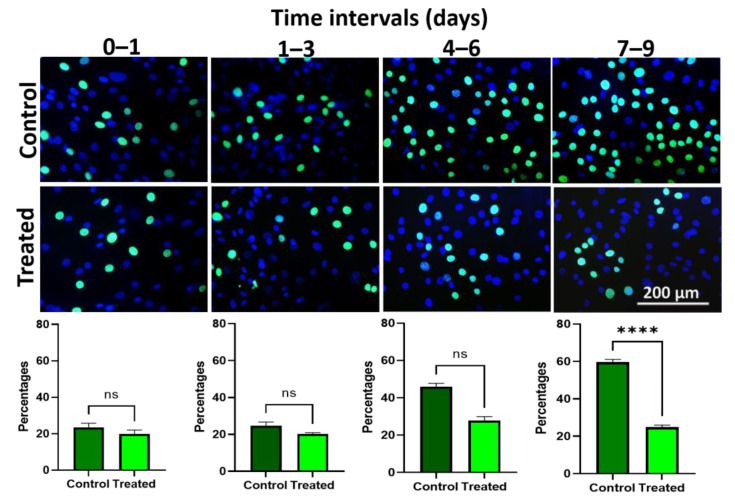
Edu proliferation assay in hLESC upon Wnt/β-catenin signaling activation. Fluorescent cytochemistry of Hoechst (blue) nuclear staining and Edu (green) staining are shown in consequent time intervals during day 1, day 1–3, day 4–6, and day 7–9 of cultivation in control hLESC cultures and treated hLESCs with 5 nM LY2090314 demonstrating lower proliferation in the treatment group after 9 days. All data from three donors (*n* = 3) are presented as mean ± SEM, ns: non-significant, ****: *p <* 0.0001.

The control hLESC cultures and hLESC cultures treated with 5 nM LY2090314 incorporated Edu during DNA replication. Edu treatment was added to the cultures during different time periods. The proliferation ratio in control vs. treated hLESC cultures was not significant during the first 24 h (control vs. treated: 23.36 ± 2.39% vs. 19.95 ± 2.11%, *p* = 0.24), from treatment day 1 to day 3 (24.79 ± 1.88% vs. 20.18 ± 0.76%, *p* = 0.09) and from day 4 to day 6 (45.88 ± 1.87% vs. 27.81 ± 2.11%, *p* = 0.1), whereas it was significant between day 7 and day 9 of treatment (59.60 ± 1.53 vs. 24.81 ± 1.14, *p* < 0.0001) (Figure 7A,B).

## 4. Discussion

### 4.1. Upon Persistent Activation of Wnt/β-Catenin Signaling the Stemness and Proliferation Decrease, Whereas the Differentiation Increases in Ex Vivo hLESC Cultures Expanded from Limbal Biopsies

Ex vivo expansion of cells from limbal biopsies is one of the most-used methods for CLET. The current setup is well suited for studying the effect of Wnt/β-catenin signaling on the self-renewal and/or differentiation status of the cells in ex vivo expanded hLESC cultures prior to transplantation. 

Previous studies have demonstrated that Wnt/β-catenin signaling activation maintains stemness and enhances proliferation in hLESC cultures isolated as single-cell cultures (without the presence of a limbal niche) [28,29,30,31,33]. In contrast, Wnt/β-catenin activation induces differentiation in hLESC cultures harvested as limbal explants (in the presence of their niche), as demonstrated by Lee et al. Our results are in line with Lee et al. since we used explants and the activation of canonical Wnt/β-catenin signaling and found reduced stemness and proliferation potential. They also explain the contrasting results from other studies using Wnt-signaling activation but using single-cell cultures instead of explants [29]. 

In embryonal stem cells, it was previously shown that Wnt/β-catenin signaling is repressed during self-renewal and that increased β-catenin induces differentiation identified by a higher expression of differentiation markers [23,34]. As explained by Lee et al., the opposite outcomes of Wnt/β-catenin activation in ex vivo cultured hLESCs by two different culture methods might be due to the difference in hLESC culture conditions. In the single-cell cultures, the hLESCs are isolated and present in small numbers, and thus the Wnt/β-catenin stimulation in this culture method was analyzed in the context of hLESC survival [28,30,31]. On the other side, Wnt/β-catenin stimulation in ex vivo expanded hLESCs out of limbal explants was observed through the context of cell ability for rapid migration and proliferation out of the limbal explants, a process that is mainly governed by TACs. Further, as the TACs divide, they obtain more differentiation properties [14,16,17]. In addition, limbal explant expansion requires rapid differentiation and leads to multilayering of the outgrowths that consist of a significant number of differentiated cells in the upper layers; these cells may then become dominant in these cell populations [35]. A similar pattern was seen in epidermal wound healing where Wnt signaling induced the differentiation of epithelial cells during epidermal wound healing [36,37]. 

The p63 marker has been for more than two decades the most-studied stemness/progenitor marker in hLESCs [38]. Later, it was revealed that only the ΔNp63α isoform is distinct for the limbal basal epithelium (LBE) where hLESCs reside, whereas other p63 isoforms were found in suprabasal layers of the limbus and cornea and may characterize the more differentiated progenies [14,39,40,41]. In contrast to our study, previous studies have demonstrated a higher proliferation rate upon Wnt/β-catenin signaling stimulation. We show a decreased proliferation potential of the hLESC cultures. It is known that hLESCs/TACs have limited proliferation potential and so, proliferation may be initially triggered and consequently exhausted upon prolonged Wnt/β-catenin stimulation [16,18,39,42]. The antiproliferative effect of selective LY2090314 has been found previously in melanoma and neuroblastoma tumors [43,44]. Our study is the first to demonstrate the effect of Wnt/β-catenin signaling activation on CEBP/δ. The *CEBPD* gene is highly expressed in the human limbus compared to the cornea itself [24]. CEBP/δ is a quiescence marker co-expressed with ΔNp63α in 10% of human LBE, but it is not present in actively dividing cells [45]. Downregulation of this marker together with p63α suggests that the Wnt/β-catenin signaling opposes stemness. The transcriptional factor SOX9 is involved during embryonal development and adult stem cell maintenance in tissues [46]. Similarly to our findings, SOX9 in hLESCs/TACs was downregulated upon Wnt signaling activation, and oppositely, upregulated by Wnt signaling inhibition. When SOX9 is knocked down, the differentiation increases in hLESCs [47]. 

The absence of Cx43 and KRT3/12 differentiation markers is also used to indicate the stem cell nature of expanded hLESCs in vitro [48]. Cx43 is a transmembrane protein involved in mammalian cell communication [49]. The absence of intercellular communication may reflect how hLESCs maintain stemness in their microenvironmental niche [13]. The lack of Cx43 punctate patterns was noted in some cells in the LBE and even more cells in the limbal epithelial crypts [13,41,50,51,52]. KRT3 and KRT12 are highly cornea-specific intermediate filaments, hallmarks of differentiated cells in the corneal epithelium [2,14]. KRT3/12 markers were absent in the LBE and limbal epithelial crypts [41,53]. 

### 4.2. Regulation of Wnt Ligands upon Wnt/β-Catenin Signaling Activation in hLESC Cultures

The activation of Wnt/β-catenin signaling was confirmed by an upregulation of the *AXIN2* gene and increased levels of total β-catenin in treated vs. untreated hLESC cultures. β-catenin has been reported to be upregulated during corneal wound healing [54], whereas *AXIN2* gene expression was found to be significantly upregulated in limbal tissue compared to the cornea [24]. This suggests an active role of Wnt-signaling in the maintenance of the corneal epithelium. We found that *WNT2* and *WNT16B* remained low and unchanged upon LY2090314 treatment, whereas the levels of *WNT6* and *WNT11* were upregulated upon Wnt/β-catenin activation in hLESCs. These four Wnt ligands are predominantly expressed in the limbal area in situ [14]. Recent research has focused on WNT6 and WNT16b ligand effects on hLESCs and found that high WNT6 and low WNT16b concentrations can increase cell maintenance and proliferation. Since they can activate both canonical and non-canonical Wnt signaling pathways in hLESC cultures, their interplay and balance can affect hLESC maintenance or differentiation processes in the limbus [14,55,56]. *WNT11* has been reported to be more specific for the limbus than the cornea itself [28]. WNT2 has been reported to be found in the superficial and suprabasal layers-, whereas WNT16 was found in the basal and suprabasal layers of the limbal epithelium and superficial layer of the corneal epithelium [57]. *WNT3* gene expression was upregulated in LY2090314 treated/Wnt/β-catenin-activated hLESCs in our study. Previously, *WNT3* was reported to be significantly higher-expressed in the central cornea compared to the limbus [28]. Inhibition of WNT3A increased SOX9 and improved holoclone formation in hLESCs [58]. Our results show *WNT7A* downregulation upon Wnt/β-catenin activation during hLESC differentiation ex vivo. Interestingly, Wnt7A is found to maintain the corneal epithelium under controlled homeostasis, prevent skin-like epithelium formation, and determine hLESC differentiation to corneal epithelial cells through PAX6 in rabbits. [59]. In line with our data, WNT7A was previously found to increase hLESC proliferation in vitro [60]. WNT5A was upregulated in our samples. WNT5A promoted cornea wound healing in rats [60] and diabetic keratopathy [61]. We report for the first time *WNT1* upregulation in hLESCs upon the activation of Wnt/β-catenin signaling.

### 4.3. Wnt Inhibitors in LY2090314 Treated and Wnt/β-Catenin Signaling Activated hLESCs

*DKK1* was significantly downregulated in the treated samples, showing enhanced differentiation compared to controls. This correlates with previous findings where Dkk1 expression stimulated hLESC pluripotency and proliferation in cultures [62]. *DKK1* was predominantly expressed in the limbus compared to the cornea [24,63]. Hence, previous studies showed that high levels of DKK1 and inhibition of Wnt/β-catenin signaling kept the hLESCs in a more pluripotent state. On the other side, downregulation of DKK1, followed by enhanced Wnt/B-catenin signaling, enhanced differentiation and loss of pluripotency in hLESCs. In our study, DKK2 expression remained stable and unchanged upon Wnt/β-catenin activation, and this was valuable since Dkk2 expression was reported to be mandatory for the proper development of the corneal epithelium in mice [64]. However, DKK inhibition in single-cell cultures affected hLESCs divergently, preserving maintenance and proliferation in small concentrations while encouraging differentiation in higher effector concentrations [31]. Similarly, the WIF1 inhibitor, predominantly expressed in the limbus [24,63], was not significantly changed in treated hLESCs in our study. Overall, none of the Wnt inhibitors were predominantly expressed in the cornea, which can indicate that DKK1 may be one of the ligands keeping the hLESC in a quiescent state in situ [59].

### 4.4. Differentially Expressed Genes Identified by Microarray Comparing hLESC Cultures Untreated or Treated with 5 nM LY2090314

Activin A receptor type 1C (ACVR1C), also known as ALK-7 (Activin receptor-like kinase 7), is a receptor binding to the ligands of the TGFβ family. ACVR1C phosphorylates directly to Smad-2/3 or Smad 1/5/8 and binds to Smad4 ligands, which then translocate to the nucleus and induce transcription [65]. ALK-7 is representative of the transforming growth factor β (TGFβ) receptor superfamily and is involved in the metabolic homeostasis of cells in many organs and tissues. However, its role in different cell types remains to be elucidated [66]. ALK-7 is known to be upregulated upon differentiation [67], and this is also in accordance with our findings, where *ALK7* is upregulated upon LY2090314 treatment of hLESCs following induced differentiation. Transforming growth factor β (TGFβ) seems to have an opposite role to Wnt/β-catenin signaling in hLESCs [68] and the balance between these two canonical pathways determines cell fate in hLESCs [62]. 

Frizzled class receptor 1 (FZD7) is found exclusively expressed in the cells of the LBE [57,63]. FZD7 is considered a possible mediator between canonical and non-canonical Wnt signaling pathway regulation in hLESCs, as shown in cancers. So far, it is known that FZD7 stimulates proliferation via the non-canonical Fzd7/syndecan4/fibronectin/ROCK pathway and the canonical β-catenin/TCF4/survival pathway [68]. The latter is in accordance with our findings since *FZD7* is upregulated upon LY2090314 treatment in addition to increased proliferation and increased levels of β-catenin in treated hLESCs. The c-Myc oncogene maintains corneal epithelial architecture at homeostasis, modulates p63 expression, and enhances proliferation during tissue repair [69].

Canonical and non-canonical Wnt ligands utilize similar systems to activate completely different cascade processes [70]. By competitional ligand binding to Fzd, canonical and noncanonical Wnt ligands exert inhibition of the corresponding pathway at the cell surface. Thus, different Wnts, through their specific coupling and phosphorylation of unrelated co-receptors, activate completely distinct signaling pathways [70]. This can also be correlated to our study in regard to the possible parallel activation of non-canonical pathways, and to what extent they affect hLESC cultures would be interesting to elaborate in further studies. The secretion of particular Wnt ligands could determine the fate of hLESCs/TACs in the limbus. Additionally, the intrinsic responsiveness of different mature stages of hLESCs/TACS to Wnt signals might be crucial for cornea homeostasis. It seems that Wnt/β-catenin signaling plays a multifunctional role in the different limbal and corneal epithelial compartments and results in a mixture of stem, progenitor, and differentiated cells. The Wnt/planar cell polarity (PCP) pathway linked with cell movement and adhesion was exclusive for late TACs and differentiated epithelial cells in the cornea [71]. In our study, the LY2090314 GSK-3 inhibitor upregulates a non-canonical WNT5A ligand, known to be able to activate or inhibit canonical Wnt/β-catenin signaling depending on the time and expression pattern of the cells [72,73]. This should be taken into consideration while interpreting our results.

Activation of the canonical Wnt/β-signaling in hLESCs was carried out through GSK-3 inhibition in our study, with the advantage of allowing the overview of spontaneous Wnt ligands and inhibitor down- and/or upregulation involved in hLESC differentiation. In contrast, previous studies have favored the Wnt/β-catenin signaling activation approach through particular Wnt ligand domination. We examined the effect of Wnt signaling activation in hLESC ex vivo cultures resulting in differentiation, reduced proliferation, and loss of stemness. This indicates the importance of regulating and avoiding activation of Wnt signaling to preserve stemness in hLESC cultures, findings similar to the dominance of the Wnt-signaling inhibitor DKK1 in the limbal stem cell niche in vivo [24]. In future research, we would like to examine the impact of inhibiting Wnt/β-catenin signaling in hLESC cultures and clarify whether this could enhance the stemness of ex vivo expanded hLESCs. The overall results of gene and protein expression in our study should be considered in light of the existence of mixed cell populations that may react diversely to Wnt/β-catenin activation. 

## 5. Conclusions

In conclusion, the present study confirms an increased differentiation and loss of stemness and proliferation upon persistent Wnt/β-catenin signaling activation with LY2090314, a GSK-3 inhibitor, in ex vivo expanded hLESCs from limbal biopsies. On the contrary, previous studies show increased stemness and proliferation upon Wnt/β-catenin signaling activation in hLESCs isolated as single-cell cultures. The comparative analysis of these two culture models evaluating Wnt/β-catenin signaling activation and inhibition would be very useful to elaborate on in future studies.

## Figures and Tables

**Table 1:** The list of genes involved in the Wnt/β-catenin canonical pathway and differentially regulated in hLESCs treated with 5 nM concentration of LY2090314 compared to the control hLESCs.

Symbol	Entrez Gene Name	*p*-Value	Fold Change
Differentially expressed genes that increase in the hLESCs treated with 5 nM LY2090314			
*ACVR1C*	Activin A receptory type 1C	3.1 × 10^−3^	2.0
*AXIN 2*	Axin 2	3.9 × 10^−6^	6.36
*FZD7*	Frizzled class receptor 7	1.3 × 10^−4^	2.24
*GJA1*	Gap junction protein alpha 1	6.2 × 10^−9^	3.66
*MMP7*	Matrix metallopeptidase 7	3.2 × 10^−5^	4.3
*PPP2R2B*	Protein phosphatase 2 regulatory subunit B beta	6.4 × 10^−5^	4.07
*TCF7*	Transcription factor 7	2.3 × 10^−4^	2.16
*TGFB3*	Transforming growth factor beta 3	3.7 × 10^−5^	2.69
*WNT5A*	Wnt family member 5A	7.4 × 10^−5^	2.28
Differentially expressed genes that decrease in the hLESCs treated with 5 nM LY2090314			
*DKK1*	Dickkopf Wnt signaling pathway inhibitor	5.5 × 10^−7^	−3.34
*MYC*	MYC proto-oncogene, bHLH transcription factor	2.2 × 10^−6^	−2.25
*SRFP1*	Secreted frizzled related protein 1	2.3 × 10^−4^	−3.76
*TGFB2*	Transforming growth factor beta 2	1.1 × 10^−3^	−2.42
*WNT7A*	Wnt family member 7A	8.3 × 10^−4^	−2.00

## Data Availability

The data that support the findings of this study are available from the corresponding author upon reasonable request.

## Supplementary Materials

The following supporting information can be downloaded at: https://www.mdpi.com/article/10.3390/biomedicines11071829/s1, Figure S1: Morphology changes in primary hLESC cultures upon dose-dependent LY2090314 treatment; Table S1: Assays used to identify changes in gene expression by real time qRT-PCR.

## References

[1] Nosrati H., Alizadeh Z., Nosrati A., Ashrafi-Dehkordi K., Banitalebi-Dehkordi M., Sanami S., Khodaei M. (2021). Stem cell-based therapeutic strategies for corneal epithelium regeneration. Tissue Cell.

[2] Schermer A., Galvin S., Sun T.T. (1986). Differentiation-related expression of a major 64K corneal keratin in vivo and in culture suggests limbal location of corneal epithelial stem cells. J. Cell Biol..

[3] Sun T.T., Tseng S.C., Lavker R.M. (2010). Location of corneal epithelial stem cells. Nature.

[4] Cotsarelis G., Cheng S.Z., Dong G., Sun T.T., Lavker R.M. (1989). Existence of slow-cycling limbal epithelial basal cells that can be preferentially stimulated to proliferate: Implications on epithelial stem cells. Cell.

[5] Dua H.S., Shanmuganathan V.A., Powell-Richards A.O., Tighe P.J., Joseph A. (2005). Limbal epithelial crypts: A novel anatomical structure and a putative limbal stem cell niche. Br. J. Ophthalmol..

[6] Goldberg M.F., Bron A.J. (1982). Limbal palisades of Vogt. Trans. Am. Ophthalmol. Soc..

[7] Townsend W.M. (1991). The limbal palisades of Vogt. Trans. Am. Ophthalmol. Soc..

[8] Schlötzer-Schrehardt U., Dietrich T., Saito K., Sorokin L., Sasaki T., Paulsson M., Kruse F.E. (2007). Characterization of extracellular matrix components in the limbal epithelial stem cell compartment. Exp. Eye Res..

[9] Tseng S.C., He H., Zhang S., Chen S.Y. (2016). Niche Regulation of Limbal Epithelial Stem Cells: Relationship between Inflammation and Regeneration. Ocul. Surf..

[10] Haagdorens M., Van Acker S.I., Van Gerwen V., Dhubhghaill S.N., Koppen C., Tassignon M.J., Zakaria N. (2016). Limbal Stem Cell Deficiency: Current Treatment Options and Emerging Therapies. Stem Cells Int..

[11] Ljubimov A.V., Saghizadeh M. (2015). Progress in corneal wound healing. Prog. Retin. Eye Res..

[12] Schlötzer-Schrehardt U., Kruse F.E. (2005). Identification and characterization of limbal stem cells. Exp. Eye Res..

[13] Matic M., Petrov I.N., Chen S., Wang C., Dimitrijevich S.D., Wolosin J.M. (1997). Stem cells of the corneal epithelium lack connexins and metabolite transfer capacity. Differentiation.

[14] Bonnet C., González S., Roberts J.S., Robertson S.Y.T., Ruiz M., Zheng J., Deng S.X. (2021). Human limbal epithelial stem cell regulation, bioengineering and function. Prog. Retin. Eye Res..

[15] Clevers H., Loh K.M., Nusse R. (2014). Stem cell signaling. An integral program for tissue renewal and regeneration: Wnt signaling and stem cell control. Science.

[16] Tseng S.C. (1989). Concept and application of limbal stem cells. Eye.

[17] Di Girolamo N. (2015). Moving epithelia: Tracking the fate of mammalian limbal epithelial stem cells. Prog. Retin. Eye Res..

[18] Lavker R.M., Tseng S.C., Sun T.T. (2004). Corneal epithelial stem cells at the limbus: Looking at some old problems from a new angle. Exp. Eye Res..

[19] Davanger M., Evensen A. (1971). Role of the pericorneal papillary structure in renewal of corneal epithelium. Nature.

[20] Deng S.X., Borderie V., Chan C.C., Dana R., Figueiredo F.C., Gomes J.A.P., Pellegrini G., Shimmura S., Kruse F.E. (2019). Global Consensus on Definition, Classification, Diagnosis, and Staging of Limbal Stem Cell Deficiency. Cornea.

[21] Pellegrini G., Traverso C.E., Franzi A.T., Zingirian M., Cancedda R., De Luca M. (1997). Long-term restoration of damaged corneal surfaces with autologous cultivated corneal epithelium. Lancet.

[22] Katoh M., Katoh M. (2007). WNT signaling pathway and stem cell signaling network. Clin. Cancer Res..

[23] Liu J., Xiao Q., Xiao J., Niu C., Li Y., Zhang X., Zhou Z., Shu G., Yin G. (2022). Wnt/β-catenin signalling: Function, biological mechanisms, and therapeutic opportunities. Signal. Transduct. Target Ther..

[24] Nakatsu M.N., Vartanyan L., Vu D.M., Ng M.Y., Li X., Deng S.X. (2013). Preferential biological processes in the human limbus by differential gene profiling. PLoS ONE.

[25] Komiya Y., Habas R. (2008). Wnt signal transduction pathways. Organogenesis.

[26] Nusse R., Clevers H. (2017). Wnt/β-Catenin Signaling, Disease, and Emerging Therapeutic Modalities. Cell.

[27] Nelson W.J., Nusse R. (2004). Convergence of Wnt, beta-catenin, and cadherin pathways. Science.

[28] Nakatsu M.N., Ding Z., Ng M.Y., Truong T.T., Yu F., Deng S.X. (2011). Wnt/β-catenin signaling regulates proliferation of human cornea epithelial stem/progenitor cells. Investig. Ophthalmol. Vis. Sci..

[29] Lee H.J., Wolosin J.M., Chung S.H. (2017). Divergent effects of Wnt/β-catenin signaling modifiers on the preservation of human limbal epithelial progenitors according to culture condition. Sci. Rep..

[30] Zhang C., Mei H., Robertson S.Y.T., Lee H.J., Deng S.X., Zheng J.J. (2020). A Small-Molecule Wnt Mimic Improves Human Limbal Stem Cell Ex Vivo Expansion. iScience.

[31] González S., Oh D., Baclagon E.R., Zheng J.J., Deng S.X. (2019). Wnt Signaling Is Required for the Maintenance of Human Limbal Stem/Progenitor Cells In Vitro. Investig. Ophthalmol. Vis. Sci..

[32] Edgar R., Domrachev M., Lash A.E. (2002). Gene Expression Omnibus: NCBI gene expression and hybridization array data repository. Nucleic Acids Res..

[33] Ma D.H., Chen H.C., Ma K.S., Lai J.Y., Yang U., Yeh L.K., Hsueh Y.J., Chu W.K., Lai C.H., Chen J.K. (2016). Preservation of human limbal epithelial progenitor cells on carbodiimide cross-linked amniotic membrane via integrin-linked kinase-mediated Wnt activation. Acta Biomater..

[34] Davidson K.C., Adams A.M., Goodson J.M., McDonald C.E., Potter J.C., Berndt J.D., Biechele T.L., Taylor R.J., Moon R.T. (2012). Wnt/β-catenin signaling promotes differentiation, not self-renewal, of human embryonic stem cells and is repressed by Oct4. Proc. Natl. Acad. Sci. USA.

[35] Szabó D.J., Noer A., Nagymihály R., Josifovska N., Andjelic S., Veréb Z., Facskó A., Moe M.C., Petrovski G. (2015). Long-Term Cultures of Human Cornea Limbal Explants Form 3D Structures Ex Vivo—Implications for Tissue Engineering and Clinical Applications. PLoS ONE.

[36] Fathke C., Wilson L., Shah K., Kim B., Hocking A., Moon R., Isik F. (2006). Wnt signaling induces epithelial differentiation during cutaneous wound healing. BMC Cell Biol..

[37] Houschyar K.S., Momeni A., Pyles M.N., Maan Z.N., Whittam A.J., Siemers F. (2015). Wnt signaling induces epithelial differentiation during cutaneous wound healing. Organogenesis.

[38] Pellegrini G., Dellambra E., Golisano O., Martinelli E., Fantozzi I., Bondanza S., Ponzin D., McKeon F., De Luca M. (2001). p63 identifies keratinocyte stem cells. Proc. Natl. Acad. Sci. USA.

[39] Dua H.S., Joseph A., Shanmuganathan V.A., Jones R.E. (2003). Stem cell differentiation and the effects of deficiency. Eye.

[40] Kawasaki S., Tanioka H., Yamasaki K., Connon C.J., Kinoshita S. (2006). Expression and tissue distribution of p63 isoforms in human ocular surface epithelia. Exp. Eye Res..

[41] Gonzalez G., Sasamoto Y., Ksander B.R., Frank M.H., Frank N.Y. (2018). Limbal stem cells: Identity, developmental origin, and therapeutic potential. Wiley Interdiscip. Rev. Dev. Biol..

[42] Dua H.S., Azuara-Blanco A. (2000). Limbal stem cells of the corneal epithelium. Surv. Ophthalmol..

[43] Atkinson J.M., Rank K.B., Zeng Y., Capen A., Yadav V., Manro J.R., Engler T.A., Chedid M. (2015). Activating the Wnt/β-Catenin Pathway for the Treatment of Melanoma—Application of LY2090314, a Novel Selective Inhibitor of Glycogen Synthase Kinase-3. PLoS ONE.

[44] Kunnimalaiyaan S., Schwartz V.K., Jackson I.A., Clark Gamblin T., Kunnimalaiyaan M. (2018). Antiproliferative and apoptotic effect of LY2090314, a GSK-3 inhibitor, in neuroblastoma in vitro. BMC Cancer.

[45] Barbaro V., Testa A., Di Iorio E., Mavilio F., Pellegrini G., De Luca M. (2007). C/EBPdelta regulates cell cycle and self-renewal of human limbal stem cells. J. Cell Biol..

[46] Jo A., Denduluri S., Zhang B., Wang Z., Yin L., Yan Z., Kang R., Shi L.L., Mok J., Lee M.J. (2014). The versatile functions of Sox9 in development, stem cells, and human diseases. Genes Dis..

[47] Menzel-Severing J., Zenkel M., Polisetti N., Sock E., Wegner M., Kruse F.E., Schlötzer-Schrehardt U. (2018). Transcription factor profiling identifies Sox9 as regulator of proliferation and differentiation in corneal epithelial stem/progenitor cells. Sci. Rep..

[48] Grueterich M., Espana E.M., Touhami A., Ti S.E., Tseng S.C. (2002). Phenotypic study of a case with successful transplantation of ex vivo expanded human limbal epithelium for unilateral total limbal stem cell deficiency. Ophthalmology.

[49] Ribeiro-Rodrigues T.M., Martins-Marques T., Morel S., Kwak B.R., Girao H. (2017). Role of connexin 43 in different forms of intercellular communication-gap junctions, extracellular vesicles and tunnelling nanotubes. J. Cell Sci..

[50] Wang I.J., Tsai R.J., Yeh L.K., Tsai R.Y., Hu F.R., Kao W.W. (2011). Changes in corneal basal epithelial phenotypes in an altered basement membrane. PLoS ONE.

[51] Shanmuganathan V.A., Foster T., Kulkarni B.B., Hopkinson A., Gray T., Powe D.G., Lowe J., Dua H.S. (2007). Morphological characteristics of the limbal epithelial crypt. Br. J. Ophthalmol..

[52] Matic M., Petrov I.N., Rosenfeld T., Wolosin J.M. (1997). Alterations in connexin expression and cell communication in healing corneal epithelium. Investig. Ophthalmol. Vis. Sci..

[53] Rodrigues M., Ben-Zvi A., Krachmer J., Schermer A., Sun T.T. (1987). Suprabasal expression of a 64-kilodalton keratin (no. 3) in developing human corneal epithelium. Differentiation.

[54] Lu R., Bian F., Zhang X., Qi H., Chuang E.Y., Pflugfelder S.C., Li D.Q. (2011). The β-catenin/Tcf4/survivin signaling maintains a less differentiated phenotype and high proliferative capacity of human corneal epithelial progenitor cells. Int. J. Biochem. Cell Biol..

[55] Clémence B., Denise O., Hua M., Sarah R., Derek C., Jean-Louis B., Francine B.-C., Sophie X.D., Jie J.Z. (2021). Wnt6 Plays a Complex Role in Maintaining Human Limbal Stem/Progenitor Cells. Sci. Rep..

[56] Zhao S., Wan X., Dai Y., Gong L., Le Q. (2022). WNT16B enhances the proliferation and self-renewal of limbal epithelial cells via CXCR4/MEK/ERK signaling. Stem Cell Rep..

[57] Mei H., Nakatsu M.N., Baclagon E.R., Deng S.X. (2014). Frizzled 7 maintains the undifferentiated state of human limbal stem/progenitor cells. Stem Cells.

[58] Peng H., Park J.K., Katsnelson J., Kaplan N., Yang W., Getsios S., Lavker R.M. (2015). microRNA-103/107 Family Regulates Multiple Epithelial Stem Cell Characteristics. Stem Cells.

[59] Ouyang H., Xue Y., Lin Y., Zhang X., Xi L., Patel S., Cai H., Luo J., Zhang M., Zhang M. (2014). WNT7A and PAX6 define corneal epithelium homeostasis and pathogenesis. Nature.

[60] Lyu J., Joo C.K. (2005). Wnt-7a up-regulates matrix metalloproteinase-12 expression and promotes cell proliferation in corneal epithelial cells during wound healing. J. Biol. Chem..

[61] Shah R., Spektor T.M., Punj V., Turjman S., Ghiam S., Kim J., Tolstoff S., Amador C., Chun S.T., Weisenberger D.J. (2021). Wnt5a promotes diabetic corneal epithelial wound healing and limbal stem cell expression. Investig. Ophthalmol. Vis. Sci..

[62] Han B., Chen S.Y., Zhu Y.T., Tseng S.C. (2014). Integration of BMP/Wnt signaling to control clonal growth of limbal epithelial progenitor cells by niche cells. Stem Cell Res..

[63] Bonnet C., Ruiz M., Gonzalez S., Tseng C.H., Bourges J.L., Behar-Cohen F., Deng S.X. (2023). Single mRNA detection of Wnt signaling pathway in the human limbus. Exp. Eye Res..

[64] Mukhopadhyay M., Gorivodsky M., Shtrom S., Grinberg A., Niehrs C., Morasso M.I., Westphal H. (2006). Dkk2 plays an essential role in the corneal fate of the ocular surface epithelium. Development.

[65] Bondestam J., Huotari M.A., Morén A., Ustinov J., Kaivo-Oja N., Kallio J., Horelli-Kuitunen N., Aaltonen J., Fujii M., Moustakas A. (2001). cDNA cloning, expression studies and chromosome mapping of human type I serine/threonine kinase receptor ALK7 (ACVR1C). Cytogenet. Cell Genet..

[66] Ibáñez C.F. (2022). Regulation of metabolic homeostasis by the TGF-β superfamily receptor ALK7. Febs J..

[67] Pedersen D.J., Guilherme A., Danai L.V., Heyda L., Matevossian A., Cohen J., Nicoloro S.M., Straubhaar J., Noh H.L., Jung D. (2015). A major role of insulin in promoting obesity-associated adipose tissue inflammation. Mol. Metab..

[68] Robertson S.Y.T., Roberts J.S., Deng S.X. (2021). Regulation of Limbal Epithelial Stem Cells: Importance of the Niche. Int. J. Mol. Sci..

[69] Portal C., Wang Z., Scott D.K., Wolosin J.M., Iomini C. (2022). The c-Myc Oncogene Maintains Corneal Epithelial Architecture at Homeostasis, Modulates p63 Expression, and Enhances Proliferation During Tissue Repair. Investig. Ophthalmol. Vis. Sci..

[70] Grumolato L., Liu G., Mong P., Mudbhary R., Biswas R., Arroyave R., Vijayakumar S., Economides A.N., Aaronson S.A. (2010). Canonical and noncanonical Wnts use a common mechanism to activate completely unrelated coreceptors. Genes Dev..

[71] Kulkarni B.B., Tighe P.J., Mohammed I., Yeung A.M., Powe D.G., Hopkinson A., Shanmuganathan V.A., Dua H.S. (2010). Comparative transcriptional profiling of the limbal epithelial crypt demonstrates its putative stem cell niche characteristics. BMC Genom..

[72] van Amerongen R., Fuerer C., Mizutani M., Nusse R. (2012). Wnt5a can both activate and repress Wnt/β-catenin signaling during mouse embryonic development. Dev. Biol..

[73] Mikels A.J., Nusse R. (2006). Purified Wnt5a protein activates or inhibits beta-catenin-TCF signaling depending on receptor context. PLoS Biol..

