# Supplementary material for: Wnt/β-Catenin Signaling Activation Induces Differentiation in Human Limbal Epithelial Stem Cells Cultured Ex Vivo
Source: Biomedicines. 2023 Jun 26;11(7):1829. doi: 10.3390/biomedicines11071829 (PMC10377110; doi:10.3390/biomedicines11071829)
Supplement: Supplementary file 1 [file biomedicines-11-01829-s001.zip › biomedicines-2401552-supplementary.pdf]

Supplementary information

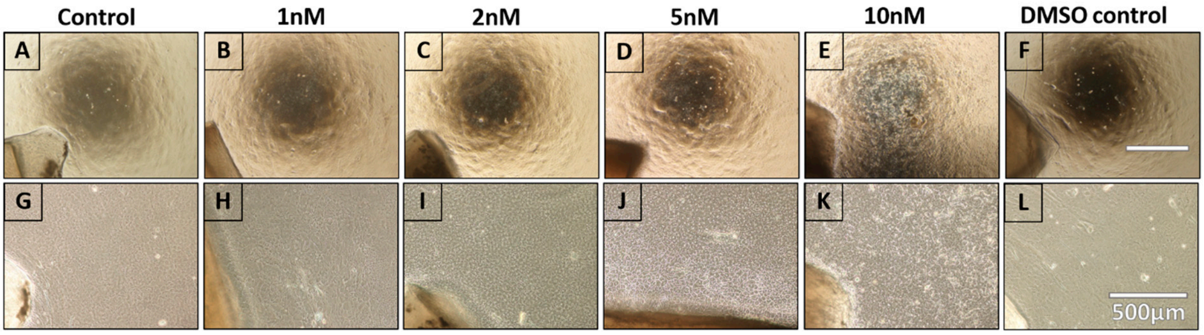

Figure S1. Morphology changes of primary hLESC cultures upon dose-dependent LY2090314 treatment. Light microscopy of the primary hLESCs growing out of limbal biopsies (left lower corner). hLESCs were cultured for nine days in COM initially, then increasing concentrations of LY2090314 were added to individual wells for six days. Control hLESCs (A, G) showed no morphological difference in comparison to DMSO control (F, L). hLESC culture appearance was altered upon 1 nM, 2 nM, and particularly 5 nM concentrations in regards to cell size, membrane thickening, and nucleus-to-cytoplasm (N:C) ratio. Cell culture treated with 10 nm LY2090314 concentration (E, K) seemed to be harmful after six days of treatment, as many damaged cells appeared in the superficial layers of the culture, and dead cells were found in the supernatants. Scale bars are the same for all images in the same row

Table S1. Assays used to identify changes in gene expression by real time qRT-PCR.

Gene name	Symbol	Taqman assay ID	Company
Tumor protein 63	TP63	Hs00978340_1	Applied Biosystems™
Marker of proliferation Ki-67	MKI67	Hs04260396_g1	Applied Biosystems™
CCAT/enhancer binding protein delta	CEBPD	Hs00270931_s1	Applied Biosystems™
SRY-Box Transcription Factor 9	SOX9	Hs00165814_m1	Applied Biosystems™
Proliferation cell nuclear antigen	PCNA	Hs00427214_g1	Applied Biosystems™
Gap junction protein alpha 1/Connexin-43	GJA1/CX43	Hs00748445_s1	Applied Biosystems™
Keratin, type II cytoskeletal 3	KRT3	Hs00365080_m1	Applied Biosystems™
Keratin, type I cytoskeletal 12	KRT12	Hs01057905_g1	Applied Biosystems™
AXIN2 / Conductin	AXIN2	Hs00610344_m1	Applied Biosystems™
Wnt Family Member 1	WNT1	Hs00180529_m1	Applied Biosystems™
Wnt Family Member 2	WNT2	Hs00608224_m1	Applied Biosystems™
Wnt Family Member 3	WNT3	Hs00902257_m1	Applied Biosystems™
Wnt Family Member 5A	WNT5A	Hs00998537_m1	Applied Biosystems™
Wnt Family Member 6	WNT6	Hs00362452_m1	Applied Biosystems™
Wnt Family Member 7A	WNT7A	Hs01114990_m1	Applied Biosystems™
Wnt Family Member 11	WNT11	Hs01045906_m1	Applied Biosystems™
Wnt Family Member 16B	WNT16B	Hs05046520_s1	Applied Biosystems™
Wnt Inhibitory Factor 1	WIF1	Hs00183662_m1	Applied Biosystems™
Dickkopf WNT Signaling Pathway Inhibitor 1	DKK1	Hs00183740_m1	Applied Biosystems™
Dickkopf WNT Signaling Pathway Inhibitor 2	DKK2	Hs00205294_m1	Applied Biosystems™
